# Genetic Signature of Histiocytic Sarcoma Revealed by a Sleeping Beauty Transposon Genetic Screen in Mice

**DOI:** 10.1371/journal.pone.0097280

**Published:** 2014-05-14

**Authors:** Raha A. Been, Michael A. Linden, Courtney J. Hager, Krista J. DeCoursin, Juan E. Abrahante, Sean R. Landman, Michael Steinbach, Aaron L. Sarver, David A. Largaespada, Timothy K. Starr

**Affiliations:** 1 Masonic Cancer Center, University of Minnesota, Minneapolis, Minnesota, United States of America; 2 College of Veterinary Medicine, University of Minnesota, St. Paul, Minnesota, United States of America; 3 Department of Comparative and Molecular Biosciences, University of Minnesota, St. Paul, Minnesota, United States of America; 4 Obstetrics, Gynecology, and Women's Health, University of Minnesota, Minneapolis, Minnesota, United States of America; 5 Department of Laboratory Medicine and Pathology, University of Minnesota, Minneapolis, Minnesota, United States of America; 6 Department of Genetics, Cell Biology, and Development, University of Minnesota, Minneapolis, Minnesota, United States of America; 7 Department of Computer Science and Engineering, University of Minnesota, Minneapolis, Minnesota, United States of America; Southern Illinois University School of Medicine, United States of America

## Abstract

Histiocytic sarcoma is a rare, aggressive neoplasm that responds poorly to therapy. Histiocytic sarcoma is thought to arise from macrophage precursor cells via genetic changes that are largely undefined. To improve our understanding of the etiology of histiocytic sarcoma we conducted a forward genetic screen in mice using the *Sleeping Beauty* transposon as a mutagen to identify genetic drivers of histiocytic sarcoma. *Sleeping Beauty* mutagenesis was targeted to myeloid lineage cells using the *Lysozyme2* promoter. Mice with activated *Sleeping Beauty* mutagenesis had significantly shortened lifespan and the majority of these mice developed tumors resembling human histiocytic sarcoma. Analysis of transposon insertions identified 27 common insertion sites containing 28 candidate cancer genes. Several of these genes are known drivers of hematological neoplasms, like *Raf1*, *Fli1*, and *Mitf*, while others are well-known cancer genes, including *Nf1*, *Myc*, *Jak2*, and *Pten*. Importantly, several new potential drivers of histiocytic sarcoma were identified and could serve as targets for therapy for histiocytic sarcoma patients.

## Introduction

Histiocytic sarcoma (HS) is classified as a neoplastic proliferation with features of histiocytes/macrophages[1]. HS has also been called true histiocytic lymphoma or malignant histiocytosis, but these terms have been discontinued. Before 1990, the majority of patients diagnosed with HS were misdiagnosed due to a lack of antibodies specific for the histiocytic lineage. Retrospective analysis indicated the majority of these patients actually had B- or T-cell lymphomas[2]–[5]. Case studies have demonstrated that HS can occur in isolation or in the context of other hematological malignancies, such as B-cell lymphoma, to which the HS is sometimes clonally related[4]. HS may thus, in some cases, develop via trans-differentiation from a malignant, or premalignant, lymphoid neoplasm. HS is rare, with an incidence far less common than the non-Hodgkin lymphomas[1], [6]. Typically, patients present with advanced clinical disease and have a poor prognosis[1], [4], [5]. Since the genetic etiology of HS is largely unknown, HS is difficult to manage clinically and there is no standard therapy for patients with HS.

Currently, no precursor lesions or etiologic agents have been described for human HS[7]. Two cytogenetic case studies identified gains in chromosome 8 in human HS[8], [9], implicating *MYC* as a HS oncogene. Animal models have identified possible driver genetic lesions. ArrayCGH performed on over 100 canine HS samples revealed an average of 30 copy number alterations per tumor[10], while a genome wide association study in Bernese Mountain Dogs identified a strong association between HS and the *MTAP-CDKN2A* locus[11]. *Pten* and *Ink4a^ARF^* are also implicated, as compound heterozygous mice develop HS and 60% of human HS examined for protein expression show a loss of PTEN, p16^INK4A^, or p14^ARF^ [12]. Several other genetic mouse models have produced HS including *Dok1/Dok2/Dok*3 triple knockout animals[13], *Cyp1b1* knockout mice[14], *p21* knockout mice[15], and *p19^ARF^/Bax* mutant mice[16]. In addition, 50% of *Cdkn2a* deficient mice infected with Moloney murine leukemia virus developed HS, which was frequently accompanied by lymphoma[17].

To identify genetic drivers of HS we performed an unbiased forward genetic screen in mice using the *Sleeping Beauty* (SB) transposon as an insertional mutagen[18]–[20]. SB is capable of both activating proto-oncogenes and inactivating tumor suppressor genes and has been used to identify genetic drivers in a variety of cancers[21]–[32]. In this study we activated SB mutagenesis using the *Lysozyme2* (*Lyz2*) promoter in a cohort of mice resulting in early mortality and a large percentage of mice developing HS. Analysis of transposon CISs identified 28 genes, including 2 miRNAs associated with HS. Several of these genes are known oncogenes and tumor suppressors including *Nf1*, *Pten*, *Myc* and *Fli1*, while many others have not been directly associated with cancer and could be potential targets for therapy.

## Methods and Materials

### Ethics Statement

All mice were bred, cared for and euthanized in accordance with the National Institutes of Health Guidelines for the Care and Use of Laboratory Animals. All experiments were approved by the University of Minnesota Institutional Animal Care and Use Committee (Protocol # 0901A56501).

### Transgenic Mice

*Lyz-Cre* mice were obtained from Jackson Laboratories (Strain name: B6.129P2-*Lyz2tm1(cre)Ifo*/J, Cat # 004781)[33]. These mice were created using a knock-in allele that has a nuclear localized *Cre recombinase* cDNA inserted into the first coding ATG of the *Lyz2* gene. This allele abolishes endogenous *Lyz2* gene function and places *NLS-Cre* expression under the control of the endogenous *Lyz2* promoter/enhancer elements.

*Rosa26-LsL-SB11* mice backcrossed to C57BL/6J were a generous gift from Adam Dupuy (University of Iowa). These mice were described previously[22].

Three strains of *T2/Onc* transgenic mice were used. The first two strains, *T2/Onc(chr1)* and *T2/Onc(chr15),* contained roughly 25 transposons resident as a concatamer on mouse chromosomes (MMU) 1 and 15, respectively[19]. The third strain, *T2/Onc2(chr4)*, contained roughly 214 transposons resident as a concatamer on MMU 4[20].

### Genotyping and Excision PCR

We isolated tail biopsy DNA using a standard phenol-chloroform extraction method. PCR was performed using primer sequences for each transgene. Primer sequences are as follows: LyzM-Cre: primer oIMR3066 5′- CCCAGAAATGCCAGATTACG- 3′, primer oIMR3067 5′- CTTGGGCTGCCAGAATTTCTC-3′, primer oIMR3068 5′- TTACAGTCGGCCAGGCTGAC-3′; T2Onc or T2Onc2: Forward 5′- CGCTTCTCGCTTCTGTTCGC-3′, Reverse 5′- CCACCCCCAGCATTCTAGTT-3′; LsL-SB11: Wild-type Forward 5′- GGAGGGGAGTGTTGCAATACCTTT-3′; Wild-type Reverse 5′- AACTCGGGTGAGCATGTCTTTAATCTAC-3′; Transgenic Forward 5′-GGCATTGGGGGTGGTGATATAAACT-3′; and T2Onc Excision PCR was performed as previously described[19] using primer sequences: Forward 5′- GGGATGTGCTGCAAGGCGAT-3′; Reverse 5′- CAAGCTATGCATCCAACGCGTT-3′.

### PCR analysis of VDJ rearrangement at *Tcrb* and *Igh* loci

DNA was isolated from eight representative tumors and control tissues from wild-type animals. For *Tcrb* analysis, two forward primers in the V locus and one forward primer in the D locus were used in conjunction with a reverse primer in the J locus. For *IgH* analysis, two forward primers in the D locus were used with a reverse primer in the J locus. Primer sequences are as follows: Vb8.2 5′-CTACCCCCTCTCAGACATCA-3′, Jb2 5′-TGAGAGCTGTCTCCTACTATCGATT-3′, Vb11.5 5′-TGCTGGTGTCATCCAAACACCTAG, Db2 5′-GTAGGCACCTGTGGGGAAGAAACT-3′, VHQ52 5′-CGGTACCAGACTGARCATCASCAAGGAC-3′, VH7183 5′-CGGTACCAAGAASAMCCTGTWCCTGCAAATGASC-3′, JH3 5′-GTCTAGATTCTCACAAGAGTCCGATAGACCCTGG-3′.

### Kaplan-Meier Analysis

Survival was examined using a Kaplan-Meier curve (Prism Software, Graph Pad) and statistically analyzed using the logrank test controlling for multiple comparisons through the Sidak method[34].

### Histopathology and Immunohistochemistry

Mice were necropsied when moribund or at 1.5 years of age, whichever came first. Lungs, heart, lymph nodes, spleen, pancreas, sternum and all abnormal tissues were removed and visually inspected for macroscopic tumors. Tissues were either fixed in 10% formalin or snap frozen in liquid nitrogen. Formalin-fixed samples underwent standard tissue processing, were paraffin-embedded, mounted and sectioned at 5 µm. Sections were adhered to glass slides by heat fixation. Slides were processed and stained with hematoxylin-eosin (HE). Immunohistochemistry was conducted with citrate-based antigen retrieval. Tissues were stained with antibodies for Mac2 and F4/80 (Cedarlane, Burlington, NC clones M3/38 and CI:A3-1), Lyz and CD3e (Dako, Carpinteria CA polyclonal), and Pax5 (Santa Cruz, Santa Cruz CA). Tissues were analyzed by a board-certified pathologist (ML, American Board of Pathology).

### Transposon insertion analysis

Genomic DNA was isolated using standard phenol-chloroform extraction and ethanol precipitation. DNA was subjected to linker-mediated PCR as previously described[23], except that primer sequences were changed to include 12 bp barcodes and Illumina HiSeq 2000 platform-specific sequences (sequences available upon request). PCR amplicons were subjected to sequencing using the Illumina HiSeq 2000 platform following manufacturer's protocol.

Sequences were mapped to the mouse genome using BOWTIE[35] using the TAPDANCE[36] bioinformatics pipeline. TAPDANCE identifies CISs based on analysis of varying genomic window sizes, tested for significance using the Poisson distribution (p<0.05) utilizing a Bonferroni correction based on number of windows examined. Based on the 1,575 unique regions, 3 insertions in an 8.9 KB window or 4 or more insertions within a 263 KB window were considered a CIS.

### qRT-PCR

RNA was extracted from 5 mg tissue with the RNeasy Minikit (Qiagen, Valencia, CA, USA). Tissues corresponded to match normal/tumor samples from liver and spleen. RNA concentrations were determined in an Epoch spectrophotometer system (BioTek, Winooski, VT, USA). 1 ug of RNA was converted to cDNA with the ABI High-Capacity cDNA Reverse Transcription Kit (#4368814) according to manufacturer conditions. Gene specific primers were designed from sequences retrieved from Genbank using Primer 3 v4.0 (http://frodo.wi.mit.edu/primer3/). All primer sequences are available upon request. Quantitative (q)PCR was carried out in an ABI 7500 system in triplicate using the FastStart Universal SYBR Green Master (Roche, Indianapolis, IN, USA) in 20 ul reactions containing 250 nm (Final concentration) for each forward and reverse primer, 5 ul of cDNA diluted mix (∼25 ng) and 10 ul of 2X SYBR Master Mix. Cycles parameters consisted in an initial denaturation step at 95° for 10 min followed by 40 cycles of amplification at 95° for 15 s and 60° for 1 min, and a dissociation step.

### Association Analysis

Frequent itemset mining was performed to find groups of insertion regions, including larger sets of three or more regions, which frequently co-occur in the tumors. This represents a branch of data mining that originates from the analysis of market basket transaction data. More specifically, frequent itemset mining is a methodology that can efficiently determine items that are frequently purchased together from a binary transaction matrix, in which rows represent different transactions by customers at a store, columns represent the different items available, and entries in the matrix indicate whether or not that item was purchased in that transaction[37], [38]. For our purposes, tumors act as transactions (rows), genes are the items (columns), and frequent itemset mining is used to determine which sets of genes co-occur together across multiple tumors.

The set of unique insertion regions produced by TAPDANCE[36] were transformed into a set of genes by mapping each insertion region to its nearest gene. Each tumor is then represented by a set of genes containing at least one insertion in that tumor, which forms a binary transaction matrix in which rows are tumors and columns are affected genes. Closed frequent itemsets (a condensed form of frequent itemset results) were then extracted from the transaction matrix using an apriori-based algorithm to produce a list of candidate gene patterns[39]-[41]. This algorithm was run with a support threshold of three, meaning that only gene patterns that co-occur in three or more tumors are considered. Some support counts were then modified to reflect the number of unique mice that had the gene pattern, rather than the number of tumors. This is to correct for similar insertion sets in tumors originating from the same mouse.

A p-value is calculated for each candidate gene pattern by modeling the support of the pattern as the test statistic. The null distribution is modeled as a binomial with the number of trials equal to the number of tumors and the probability of success equal to the joint probability of the individual genes in the patterns occurring together (based on their individual frequencies in the dataset). In order to account for multiple hypotheses testing, the significance of each candidate pattern was determined by empirically estimating its q-value[42], which is the minimum False Discovery Rate (FDR) at which the test may be called significant[43]. Specifically, a set of 10,000 simulated results were generated by randomizing the tumor that each insertion appears in while preserving the overall set of insertion locations and the number of insertions in each tumor. The q-value for each candidate pattern was calculated as the percent of simulated results that had a p-value better or equal to the p-value of the candidate pattern divided by the percent of real patterns with a p-value equal to or better than the candidate pattern. The gene patterns with q-value ≤25% were deemed significant.

### Statistics

The Cancer Gene Census and the COSMIC database were downloaded on 4/20/2013 from Sanger Institute website (ftp://ftp.sanger.ac.uk/pub/CGP/cosmic/data_export). A custom Perl script was used to extract haematopoietic_and_lymphoid_tissue mutations from the CosmicMutantExportIncFus_v64_270313.tsv file. The list of mutations in AML were derived from supplemental tables in the TCGA report on AML[44] combining the list of Tier 1 mutated genes with the list of fusion genes. Significance of association was computed using a 2-tailed Fisher's Exact Test[45]. MAPK pathway significance was determined by performing 10,000 iterations of randomly assigning mouse genes to libraries and calculating number of libraries with insertions in MAPK pathway genes using a custom perl script.

## Results

### SB Insertional Mutagenesis Promotes Histiocytic Sarcoma Formation

To perform a forward genetic screen for HS we generated mice harboring three elements required for activating SB mutagenesis in myeloid lineage cells. The first element was a nuclear localized *Cre recombinase* gene knocked into the myeloid-specific *Lyz2* locus[46] (Fig S1-A). The *Lyz2* promoter is expressed in granulocytes, macrophages, and splenic dendritic cells[33], [47]. The second element was a conditional *SB11 transposase* allele created by inserting a *Lox-STOP-Lox-SB11-cDNA* construct downstream of the ubiquitous *Rosa26* promoter (Fig S1-B)[22], [23]. The third element was a concatamer of oncogenic SB transposons (*T2/Onc*). The SB transposon consists of terminal inverted and direct repeats required for SB transposition and an internal promoter, splice donor, splice acceptors and bidirectional polyA signal. The transposon was designed to be capable of overexpressing or disrupting genes, and these transposon-induced mutations provide cells with a selective advantage when they occur in oncogenes or tumor suppressors, respectively (Fig S1-C). The internal promoter within the transposon is highly active in hematopoietic stem cells[48]. We have shown that the SB transposon system is capable of generating insertional mutations leading to overexpression of oncogenes, overexpression of truncated genes, and disruption of genes [19], [20], [23], [49]. We designed a breeding scheme (Fig S2) and generated 73 experimental mice carrying all three elements and 117 littermate controls carrying only two of the three elements (Table S1).

**Figure 1:**
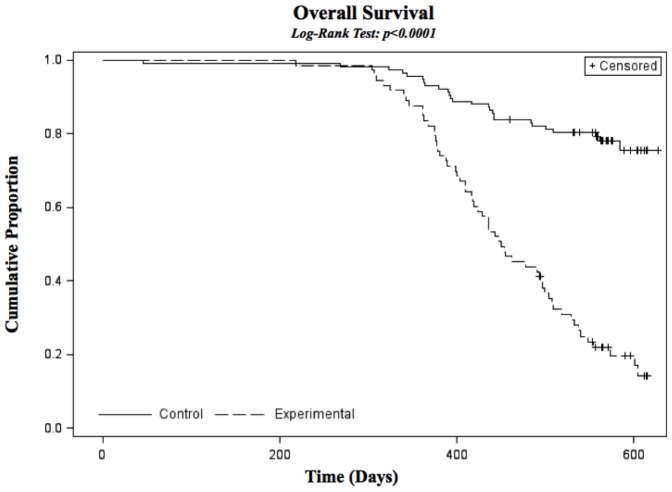
Kaplan Meier Survival Curve showing decreased survival in triple transgenic experimental animals compared to double transgenic controls. Significance determined using Logrank test.

**Figure 2:**
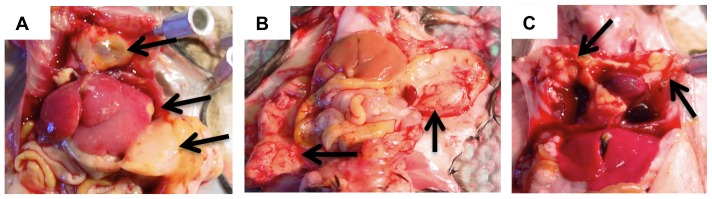
Representative images of tumor tissue. A) Disseminated HS in pancreas, liver, and thoracic cavity. B) HS adhering to peritoneum. C) HS within the thoracic cavity.

Mice were sacrificed and necropsied when they became moribund or at 18 months, whichever came first. Triple transgenic mice became moribund at a faster rate than controls, beginning around one year of age (Fig 1). The majority of mice had malignancies occurring in multiple tissues throughout the mouse (Fig 2). Over 75% of mice examined had symptoms of disease with the majority being localized to spleen, pancreas, liver, thoracic cavity and peritoneum.

**Figure 3:**
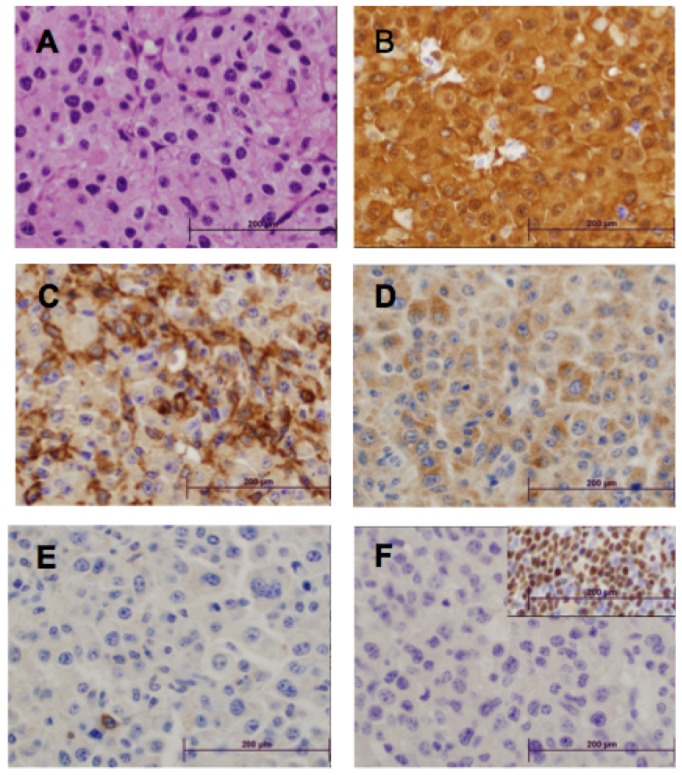
Typical morphologic and immunophenotypic characteristics of the murine histiocytic neoplasms generated by a forward genetic screen. All images were captured using a 50X oil objective. The depicted neoplasm was present near the pancreas in one mouse (see supplementary figures for additional morphologic characterization). A) H&E – note abundant granular cytoplasm and large nuclei; B) MAC2 immunostain; C) F4/80 immunostain; D) Lysozyme immunostain; E) CD3 immunostain – single lymphocyte in lower left quadrant stains positively; F) PAX-5 – insert denotes on-slide positive control.

**Figure 4:**
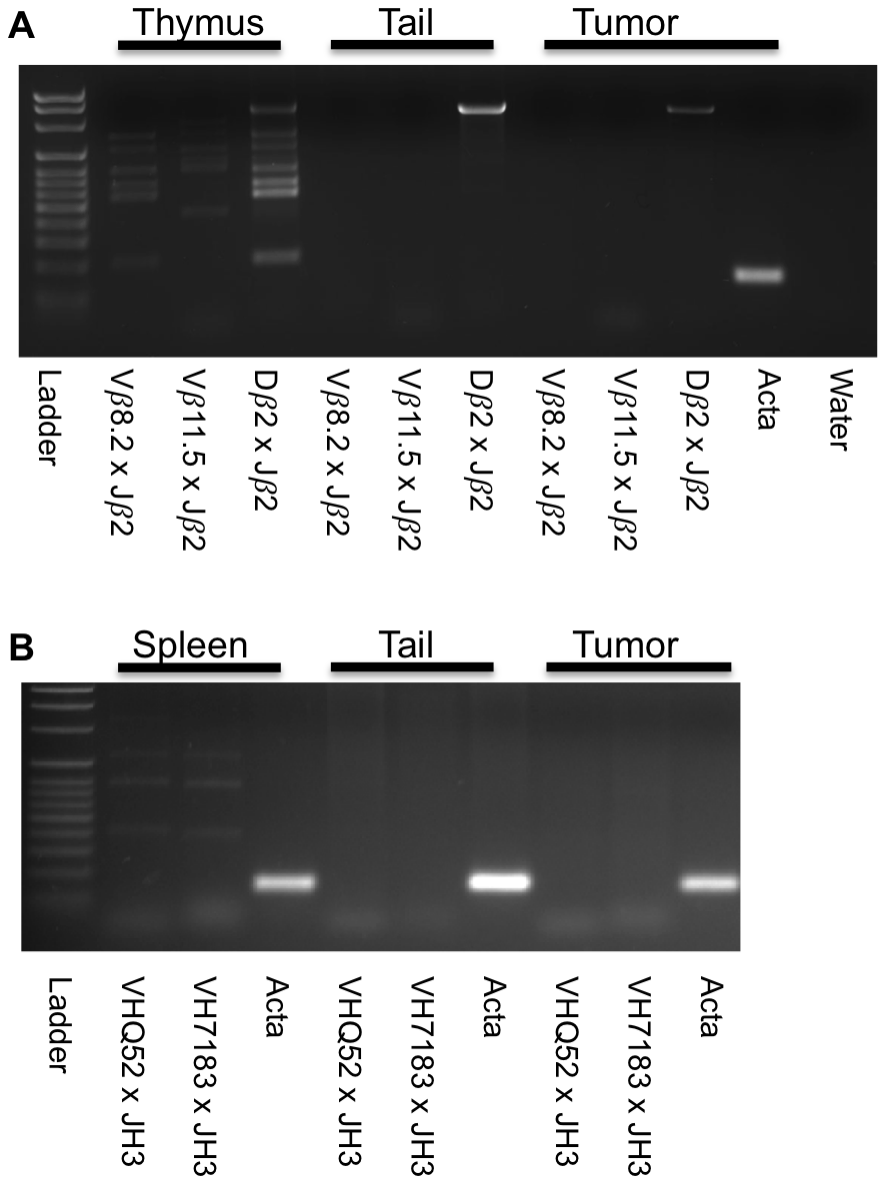
TCR and Ig genes are not rearranged in tumors. A) PCR amplification of TCR locus using genomic DNA from HS tumor (Lyz-728) indicates no rearrangement of TCR VDJ locus. B) PCR amplification of the IgH locus indicates no rearrangement of IgH DJ locus. Thymus, spleen, and tail DNA were from a wild-type control animal.

To classify the disease we prepared hematoxylin and eosin stained slides from multiple tissues from 51 animals (Fig 3A and Fig S3). Evidence of histiocytic neoplasm was visible in 33 of 51 mice (65%). Upon examination of the neoplasms by light microscopy, the tumors comprised a diffuse relatively non-cohesive proliferation of large cells. The neoplastic cells were large, round to oval in shape, with focal spindling, with large nuclei and abundant cytoplasm. The cytoplasm was eosinophilic, with fine granularity. The nuclei had vesicular chromatin, and many had prominent nucleoli. It is notable that some of the neoplasms have rather bland morphology, while others have marked pleomorphism and increased mitotic activity (Fig S3, panel G). The neoplasms invaded surrounding adjacent tissue, including muscle, spleen, liver, pancreas, lung, and bowel (Fig S3). Eight of these tissues were further analyzed by immunohistochemistry using a panel of antibodies to further confirm histiocytic differentiation (Mac2, F4/80 and Lyz) and exclude B-lineage (Pax5) or T-lineage (CD3) cells (Table S2 and Fig 3B-F). All eight tissues were strongly positive for Mac2, positive for Lyz, and negative for Pax5. Seven of the eight stained positive for F4/80, while three of eight were weakly positive for CD3; the level of CD3 staining was negligible in two of these and not diagnostic of T cell lineage. The immunophenotypic characteristics of these neoplasms in conjunction with the morphologic features are most consistent with the characteristic HS that occur in mice[7]. We also performed PCR on DNA from these same eight tumors using primers crossing VDJ boundaries in both the *TCRb* locus and the *IgH* locus. Multiple bands were amplified in control tissues (Thymus for *TCRb* and spleen for *IgH* locus) while no bands, or only germline bands were amplified in seven of eight HS tumors (representative images in Fig 4). The morphologic, immunophenotypic and molecular data support that the neoplasms are histiocytic in origin and do not have associated B- or T- lymphoid differentiation. Thus, they are best characterized as HS.

### Identification of candidate driver genes and pathways in HS

To find genetic drivers of HS we analyzed transposon insertions in 92 tumors from 36 different mice. The tumors were distributed among eight different anatomical locations (Table S3). We were able to confirm that 35 of the 92 tumors were HS based on histology. The remaining tumors are assumed to be HS based on gross pathology, but we did not have enough tissue to confirm by histological examination. We performed linker-mediated PCR (LM-PCR) on purified DNA from these tumors to amplify transposon-genomic fragments and then sequenced the amplicons using the Illumina HiSeq 2000 platform. Sequences were analyzed using a bioinformatics pipeline we developed called TAPDANCE[36]. Approximately 13.8 million sequences were mapped to the genome. Redundant sequences and sequences mapping within 100 bases of each other were combined, resulting in 11,885 non-redundant mapped regions. The depth of sequence reads using the Illumina platform allowed us to filter regions based on the number of sequence reads that mapped to the region. We reasoned that regions with only one or a few reads could either be artifacts or only present in a minority of cells, while regions with a larger number of reads were more likely to be present in a majority of tumor cells. We set a read threshold of 0.01% of total reads mapping in a single tumor for each region. For example, one of our tumors had 227,882 reads in 365 regions. Using our threshold, a single region would require at least 23 mapped reads to be included in our analysis. Of the 365 regions mapping in this tumor, only 90 met the threshold. Out of the 11,885 non-redundant regions, 1,575 unique regions met the threshold (Table S4). A BED formatted version of the unique regions (Table S5) is also provided for use with the Integrated Genome Viewer (IGV) or for uploading to a genome browser to analyze insertion positions relative to exons. This works out to approximately 17 insertions per tumor, with a range of 1 to 90.

In previous screens we noted that transposon insertions mapping to the donor chromosome, where the original transposon transgene was located, constituted up to half of all the mapped transposon insertions[19], [23], [26], a phenomenon referred to as “local hopping”. In this experiment we generated experimental mice using three different founder strains with the donor concatamer on different chromosomes in each of the strains (chr1, chr4 and chr15). Surprisingly, in these tumors, we did not see a large bias of insertions in the donor concatamer. In general, the percentage of insertions on the donor chromosome for each of the three respective T2/Onc strains was 2 to 3 times higher than expected (Table S6) Because the insertion distribution was not heavily skewed towards the donor chromosome we performed four separate CIS analyses. The first three analyses eliminated the donor chromosomes (1, 4 & 15) in those respective tumors, while the fourth analysis included all chromosomes. Of the seven CISs identified on the donor chromosomes, five of the seven were still identified in the analyses even when the insertions in those chromosomes were excluded from the subset of tumor libraries with the corresponding donor concatamer. The other two CISs (Bach2 and Atp6v1c1) were not identified if the donor chromosome was excluded, indicating they may be biased by the donor concatamer. All of the CISs identified in the four analyses were merged into a single list resulting in a final list of 27 CISs (Table 1).

**Table 1:** Common transposon insertion sites in HS tumors.

Candidate Gene	Entrez GeneID	Chr	Start	End	Predicted effect	# Mice	# Unique Regions in CIS
*Bach2* *	12014	chr4	32237300	32487700	Gain	12	10
*Raf1* *	110157	chr6	115645700	115696200	Gain	12	8
*Fli1* *	14247	chr9	32467900	32521100	Gain	8	6
*Mitf* *	17342	chr6	97865300	98065300	Gain	6	5
*No_gene_chr2*	NA	chr2	98662700	98675200	Unknown	3	5
*Pi4ka*	224020	chr16	17107400	17307400	Loss	5	4
*Sin3a*	20466	chr9	57093200	57113900	Gain	5	4
*Pten*	19211	chr19	32781300	32831300	Loss	4	4
*Orai1*	109305	chr5	123014300	123026800	Loss	6	3
*Nf1* *	18015	chr11	79422000	79472000	Loss	4	3
*Jak2*	16452	chr19	29157700	29357700	Gain	4	3
*A330023F24Rik, Mir29b-2, and Mir29c*	320977	chr1	194982400	195032400	Gain	3	3
*Rreb1* *	68750	chr13	37832600	37932600	Gain	3	3
*Rpgrip1*	77945	chr14	52114100	52314100	Unknown	3	3
*Atp6v1c1* ^†^	66335	chr15	38680400	38705400	Loss	3	3
*Pvt1*	19296	chr15	62185400	62385400	Unknown	3	3
*Kctd5*	69259	chr17	24052300	24152300	Unknown	3	3
*Il20rb*	213208	chr9	100256900	100456900	Unknown	3	3
*Kdm6a*	22289	chrX	18208700	18258700	Loss	3	3
*Ncoa2*	17978	chr1	13306200	13318700	Gain	7	2
*Serpinf1*	20317	chr11	75412500	75437500	Gain	5	2
*Myc*	17869	chr15	61877000	62077000	Unknown	5	2
*Stim1*	20866	chr7	102313500	102338500	Gain	4	2
*Xpr1*	19775	chr1	155265800	155365800	Loss	3	2
*2310035C23Rik*	227446	chr1	105754300	105766800	Loss	3	2
*Arhgap18*	73910	chr10	26851600	26876600	Loss	3	2
*Erg*	13876	chr16	95406500	95419000	Gain	3	1

* Identified as CIS in the subset of tumors with confirmed HS histopathology.

† Only identified when libraries with donor concatamer are included.

Because we could not positively diagnose all the tumors we sequenced via histology we performed a second analysis of CISs using only those tumors that had corresponding histological analysis confirming HS. Because there were fewer tumor libraries, only six CISs in this analysis were identified based on our criteria described above. All six of these CISs (*Raf1, Mitf, Nf1, Fli1, Bach2, and Rreb1*) were also present in the list of 27 CISs identified in the original analysis (Table 1).

To determine the clonality of tumors arising in a single animal we measured the overlap between tumors from the same animal. It was apparent that several tumors were clonal, based on the large percentage of shared insertions, although the majority of tumors did not share transposon insertions with tumors from the same animal (Table S7). To eliminate the bias these clonal tumors may have contributed to calculating CISs we required that all CISs consist of tumors from at least three separate mice. As a conservative test, we re-calculated CISs, this time considering all the tumors from each animal as a single tumor. This re-calculation still identified 24 of the 27 loci, indicating tumor clonality did not significantly affect CIS detection.

Manual analysis of the transposon insertion patterns in the 27 genomic loci allowed us to identify 28 candidate genes, including two micro-RNAs, and we could predict the effect (gain- or loss-of-function) for 21 of these genes based on the location and direction of the transposon insertions in the gene locus (Table 1). The three top hits, ranked by percentage of tumors contributing to the CIS, were *Raf1* (alias *C-Raf*), *Bach2* and *Fli1*. Over 25% of all tumors had a mutation in one of these three genes, and half of these tumors had mutations in at least two of the genes.

To measure the effect of the SB transposon insertions we selected a small subset of the tumors where we had sufficient frozen tumor tissue along with a matched normal tissue to extract RNA and perform qRT-PCR. We selected four tumors from three mice and measured the expression level of four genes (Fli1, Nf1, Mitf, and Raf1) in the tumors that had insertions in these four genes. Based on the transposon insertion pattern we predicted that Fli1, Mitf, and Raf1 would have gain of function mutations, while Nf1 would have a loss of function. Ten of the eleven comparisons possible in this set of tumor/normal tissue pairs indicated that the mRNA level changed in the predicted manner (Fig S4).

### Network analysis and relevance to human HS

Because *Raf1* was the site of transposon mutagenesis in over 20% of tumors analyzed, we checked for transposons inserted near other MAPK pathway genes in tumors without a *Raf1* insertion. We found that 44 of the 92 tumors (48%) generated in our screen had a transposon insertion within 10 kb of an annotated MAPK pathway gene based on the KEGG[50] MAPK pathway gene list (Table S8). To measure the significance of this finding we analyzed randomly generated datasets. The average number of libraries with a MAPK insertion, from 10,000 randomly generated datasets, was 13 (st. dev. 3.0), which is significantly lower than the 44 libraries found in our set of tumors.

To identify cooperating mutations we tested for associations between CISs using Fisher's Exact Test. After correcting for multiple testing, we found significant associations between *Fli1* and *Bach2* and between *Mitf* and *Raf1*, suggesting these pairs of mutations may cooperate in HS tumorigenesis, although formal proof of cooperation would require further experiments. Interestingly, *MITF* is an oncogene in melanoma, and leads to cell survival via upregulation of *BCL2* and other molecules[51]. This suggests the combination of a growth factor mutation and a cell survival mutation may be crucial for HS development.

We analyzed the overlap between our gene list, from which we could identify 25 human orthologs, and known human cancer genes. Ten of the 25 CIS human orthologs were in the list of 487 cancer genes annotated in the Sanger Institute's cancer gene census[52] (Table S9). This is a significant overlap (Fisher's Exact Test p<0.00001). Although all of the 25 CIS human orthologs had multiple documented somatic mutations in the COSMIC database[53], the significance of this comparison is difficult to ascertain, as over 96% of the ∼24,000 genes contained in COSMIC have documented mutations. If we limit our analysis to the 4,682 genes mutated in tumors classified as hematopoietic and lymphoid tissue in COSMIC, we find an overlap of 13 of our 25 CIS genes (Fisher's Exact Test, p<.001) (Table S9). There are no HS tumors documented in the COSMIC database. We also compared our gene list to the recent TCGA sponsored study of AML, because both HS and AML derive from the myeloid lineage. The AML study analyzed mutations and gene-fusions in 200 patient samples and identified 2,022 genes with mutations or gene fusions predicted to alter protein sequence[44]. Eleven of our 25 CIS genes overlapped with these AML genes (Table S9), which would not be expected by chance (Fisher's exact test, p<0.00001). These results support the hypothesis that our mouse model has discovered cancer genes relevant to human cancer, and myeloid malignancies specifically.

We analyzed networks associated with the 27 CIS human orthologs, including the two microRNAs using Ingenuity Pathway Analysis (Ingenuity Systems, www.ingenuity.com). Six of the top ten canonical pathways associated with our gene set were cancer signaling (Table S10), while seven of the top ten functions involved death or proliferation of cancer cells (Table S11). The major proteins contributing to these associations were *MYC, RAF1, JAK2*, and *PTEN*. These findings suggest that agents that target these signaling pathways, such as ruxolitinib or sorafenib, could be effective in HS patients with a matching genetic profile.

Finally, we used a method of identifying cooperating mutations in our tumors that does not rely on defined CISs. Instead, we used an algorithm called frequent itemset mining[37], [38]. The algorithm identifies combinations of insertions that frequently co-occur in multiple tumors. These groups of genes can reach statistical significance, even though they do not reach significance as a CIS. Analysis of 1,575 transposon insertions (Table S4) using frequent itemset mining identified 38 sets of genes that were mutated in three or more mice, with an FDR≤0.25 (Table S12). A total of 28 genes comprise the 38 sets, with several genes appearing in multiple sets. The majority of the gene sets (24/38) contained three or more of the following genes: *Pcf11, Dennd2c*, *Serpinf1, Ncoa2*, *Dctn4*, *Kif2c, Basp1* and *Raf1*. For example, three mice had tumors with transposon insertions in seven of these eight genes (See itemset #11 in Table S12). These results suggest that combinations of alterations in these genes may function coordinately to generate HS, although functional validation will require further experiments.

## Discussion

HS is a rare human neoplasm that is difficult to diagnose and has a poor prognosis. To understand the genetics of HS, with the goal of expanding treatment options for these patients, we conducted a forward genetic screen in mice using the Sleeping Beauty DNA transposon as a mutagen. The majority of mice in the experimental cohort developed symptoms associated with HS.

CIS analysis identified 26 mouse protein-coding genes and two microRNAs that are putative drivers of HS in our model. We identified human orthologs for 25 of the genes, including both microRNAs. These candidate HS cancer genes were significantly enriched for human cancer genes based on the Sanger Institute's cancer gene census and COSMIC database. The list was also enriched in genes mutated in AML based on TCGA data. The significant overlap between genes identified in our screen and known human cancer genes suggests these genes are highly relevant as candidate cancer genes in HS.

The top three genes identified in our screen have been linked to human cancers. *Raf1* is part of the MAP kinase pathway and is important for cell fate decisions. Altered *RAF1* is associated with the development of Noonan and LEOPARD syndrome, AML, and pilocytic astrocytoma[54]-[56]. *Fli1* is an ETS transcription factor and human *FLI1* forms a fusion with *EWS* in 85% of Ewing sarcoma patients. Interestingly, the other major *EWS* fusion partner found in Ewing sarcoma patients is *ERG*, another gene identified in our screen[57]. *FLI1* fusions have also been found in prostate cancer[58] and abnormal *FLI1* expression in AML patients correlates with poor prognosis[59]. *BACH2*, paradoxically, is a suspected tumor suppressor in CML and Burkitts lymphoma[60], [61]. *BACH2* is activated by oxidative stress and can inhibit proliferation and trigger apoptosis in cell lines[62]. Based on our screen we predict *Bach2* is overexpressed in HS tumors, suggesting oncogenic activity in these tumors via aberrant activation of this transcriptional repressor in myeloid cells. In support of this hypothesis, *BACH2* is significantly overexpressed in CLL and B-cell ALL[63]. Intriguingly, there are quite a few case reports of HS developing as a secondary cancer and/or morphologic variant in patients with B-cell lymphoma with evidence that the neoplasms are clonally related[64]-[66] suggesting similar genetic etiologies. BACH2 has recently been shown to be important for B-cell germinal center formation, where B cells undergo somatic hypermutation and extremely rapid proliferation[67]. It is possible that Bach2 overexpression in HS results in a transcriptional change that favors rapid proliferation in these cells.

Identifying effective targeted therapies for rare cancers is extremely difficult because it is impossible to conduct informative clinical trials due to the small number of patients. Our mouse model can be used to identify potential therapeutic targets in HS. Both *Raf1* and *Myc* were identified as candidate genes in our study. Two case reports of cytogenetic analysis of human HS have identified extra copies of chromosome 8, where *MYC* resides, suggesting *MYC* is involved in human HS[8], [9]. We found that over 50% of tumors in our screen had transposon insertions near MAPK pathway genes, suggesting that MAPK pathway inhibitors or HDAC inhibitors, like FK228, that significantly decrease RAF1 levels[68] may be effective therapeutics for HS patients.

Another possible therapeutic target for HS patients, based on our findings, is *FLI1* signaling. Abnormal expression of *FLI1* is associated with AML and T-cell lymphoma[20], [59], while *FLI1* fusion proteins are linked to Ewing sarcoma and prostate cancer[58], [69].

The “hallmarks of cancer” paradigm[70] posits that multiple pathways are disrupted in a single cancer. We used frequent itemset mining analysis of transposon insertions to identify multiple genes that were co-mutated in several tumors. This analysis identified 38 gene sets comprised of 28 genes. Analysis of these 38 gene sets indicates that different subsets of only eight genes heavily contribute to a majority of the itemsets (*Pcf11, Dennd2c*, *Serpinf1, Ncoa2*, *Dctn4*, *Kif2c, Basp1* and *Raf1*). Based on the function of these eight co-occurring genes[71]–[78] we hypothesize that the combination of effects listed in Table 2 can cooperate to generate HS. The next step will be to directly test these combinations using in vitro and in vivo models where the set of genes are coordinately manipulated and the effect on cancer phenotypes is measured.

**Table 2:** Eight candidate cooperating genes in HS.

Gene	Predicted functional effect of transposon insertion	Reference
*Pcf11*	Gain: Increased transcription termination	69
*Serpinf1*	Loss: Relief from angiogenesis inhibition	71
*Dennd2c*	Unknown: Disruption of Rab9a signaling	70
*Kif2c*	Loss: Altered chromosomal segregation	72
*Raf1*	Gain: Activation of MAP kinase signaling	73
*Dctn4*	Unknown: Altered trafficking along microtubules	74
*Ncoa2*	Gain: Altered nuclear hormone signaling	75
*Basp1*	Unknown: Altered WT1 transcription	76

In conclusion, we have identified several candidate genetic drivers of HS using a transposon-based forward genetic screen in mice. The genes we identified are frequently associated with human cancer, including cancers highly related to HS. These findings lay the groundwork for testing new therapeutics to treat this rare neoplasm that currently has a very poor prognosis.

## Supporting Information

Figure S1 **Three elements for activating SB transposition in myeloid cells.**
(TIF)

Figure S2 **Breeding scheme for generating experimental animals and littermate controls.**
(TIF)

Figure S3 **Histiocytic neoplasms (HN) in multiple mice demonstrate involvement at multiple sites, local invasion/destruction, and aggressive morphology.**
(TIF)

Figure S4 **Change in mRNA levels of genes with transposon insertions comparing tumor to matched normal tissue.**
(TIF)

Table S1 **Number and genotype of cohorts.**
(XLSX)

Table S2 **IHC scoring for 5 markers in 8 tumors.**
(XLSX)

Table S3 **List of tumors sequenced for transposon insertions.**
(XLSX)

Table S4 **Non-redundant genomic regions containing transposon insertions (Excel version).**
(XLSX)

Table S5 **Non-redundant genomic regions containing transposon insertions (BED formatted text version).**
(BED)

Table S6 **Insertion distribution by donor chromosome.**
(XLSX)

Table S7 **Clonality of multiple tumors from the same mouse based on insertion region overlap.**
(XLSX)

Table S8 **Tumors with transposon insertions in/near MAPK pathway genes.**
(XLSX)

Table S9 **CIS annotations.**
(XLSX)

Table S10 **IPA Canonical Pathways.**
(XLSX)

Table S11 **IPA Annotated Functions.**
(XLSX)

Table S12 **Coordinately mutated genes based on frequent itemset mining.**
(XLSX)
