# Supplementary figures and images for: Genetic Signature of Histiocytic Sarcoma Revealed by a Sleeping Beauty Transposon Genetic Screen in Mice
Source: PLoS One. 2014 May 14;9(5):e97280. doi: 10.1371/journal.pone.0097280 (PMC4020815; doi:10.1371/journal.pone.0097280)

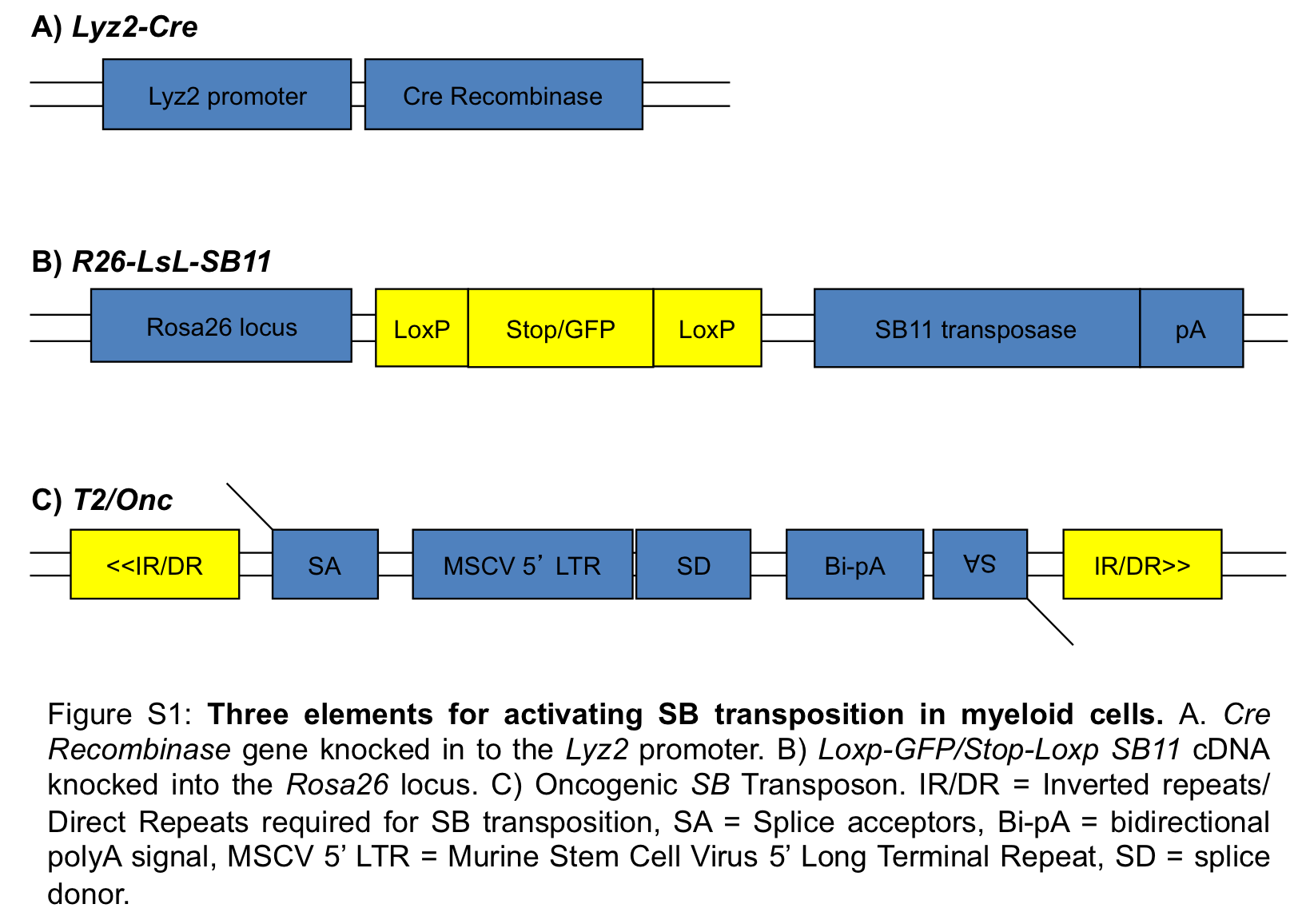

Supplement: Figure S1 — Three elements for activating SB transposition in myeloid cells. (TIF) [file pone.0097280.s001.tif]

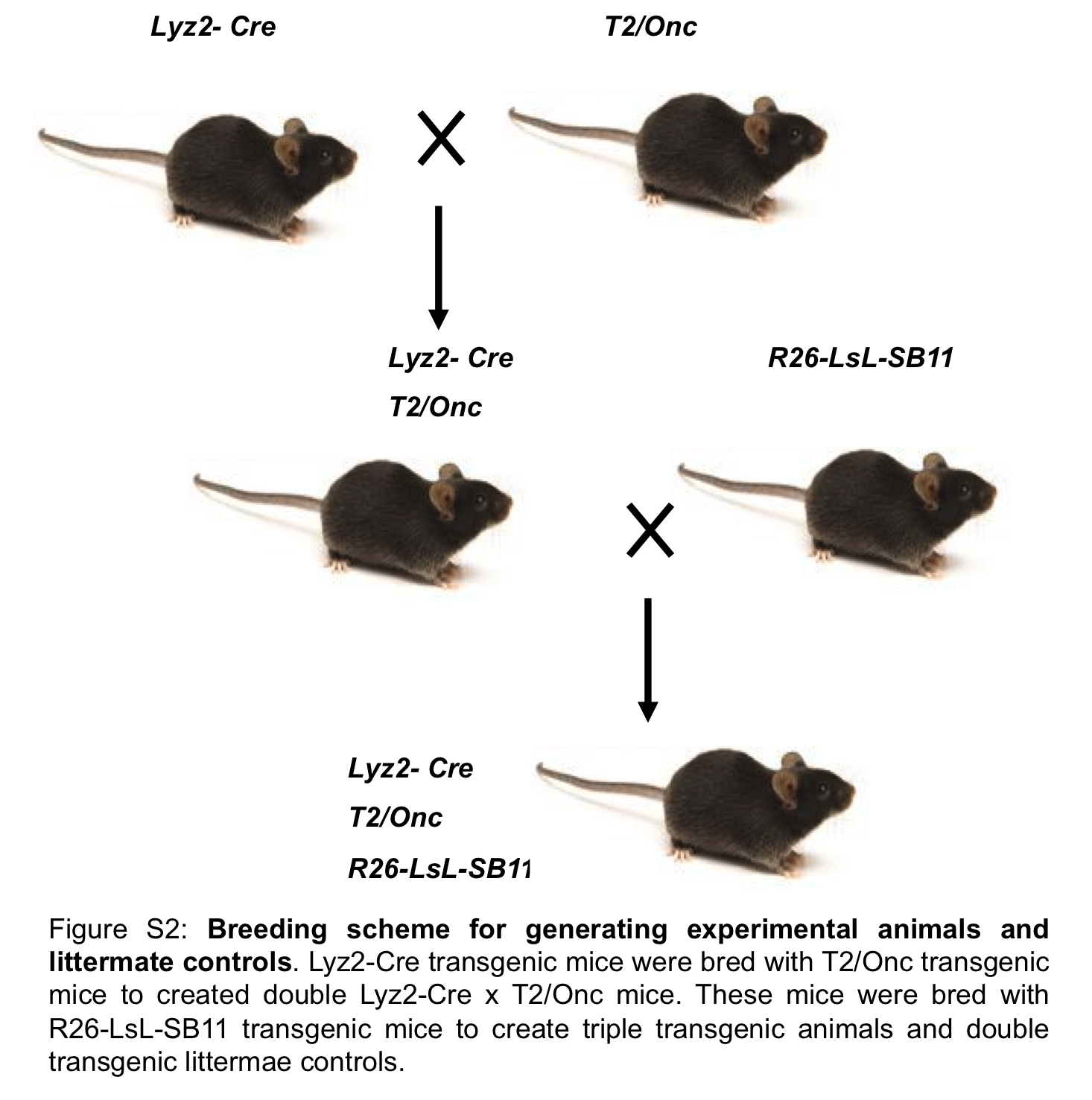

Supplement: Figure S2 — Breeding scheme for generating experimental animals and littermate controls. (TIF) [file pone.0097280.s002.tif]

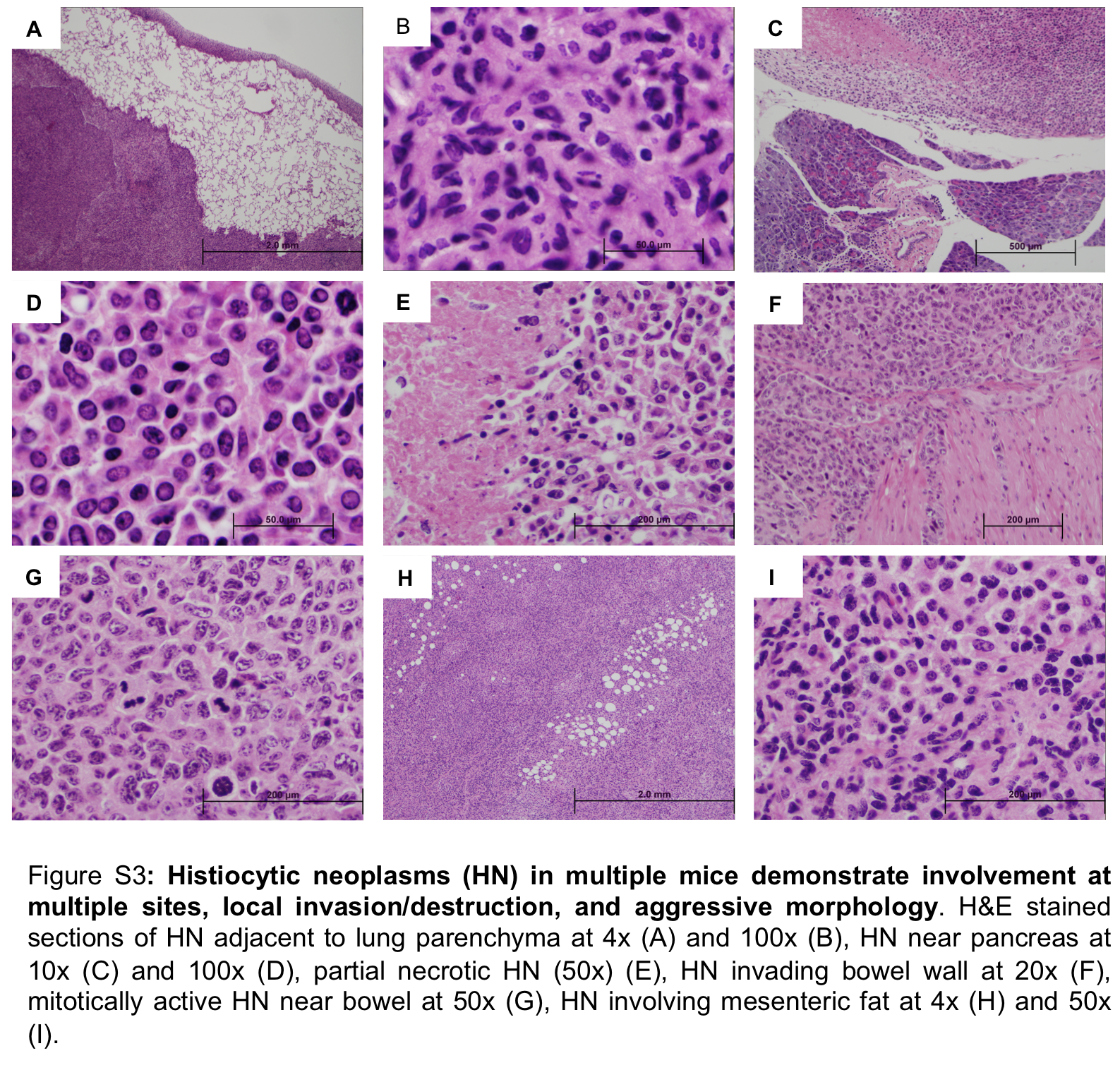

Supplement: Figure S3 — Histiocytic neoplasms (HN) in multiple mice demonstrate involvement at multiple sites, local invasion/destruction, and aggressive morphology. (TIF) [file pone.0097280.s003.tif]

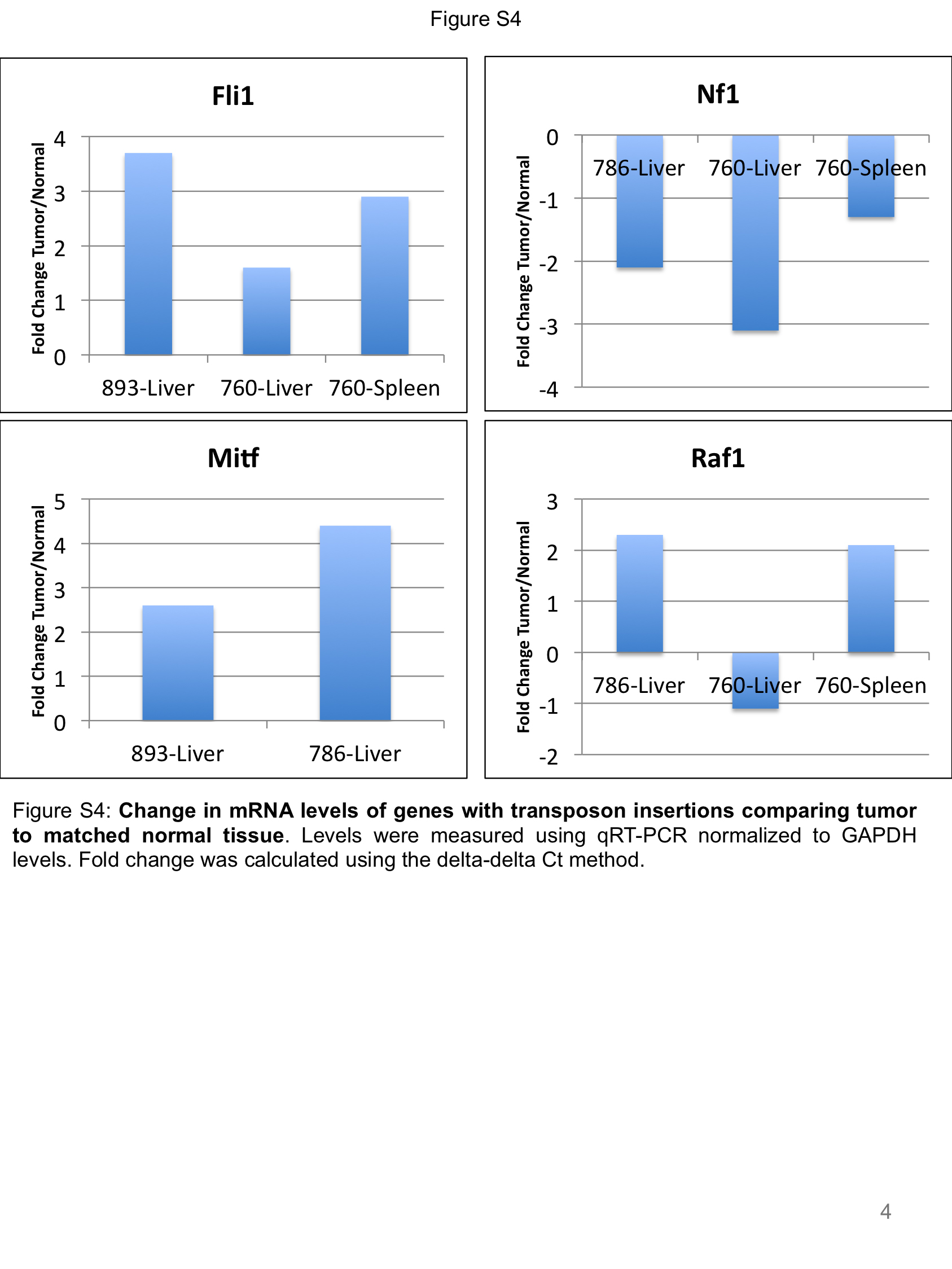

Supplement: Figure S4 — Change in mRNA levels of genes with transposon insertions comparing tumor to matched normal tissue. (TIF) [file pone.0097280.s004.tif]
